# *In vitro* and *in vivo* development of mice morulae after storage in non-frozen conditions

**DOI:** 10.1186/1477-7827-10-62

**Published:** 2012-08-22

**Authors:** Juan de Dios Hourcade, Miriam Pérez-Crespo, Alfredo Serrano, Alfonso Gutiérrez-Adán, Belén Pintado

**Affiliations:** 1 Dpto. de Reproducción Animal, INIA, Ctra de la Coruña Km 5,9, Madrid, 28040, Spain; 2 Centro Nacional de Biotecnología, CSIC. C/ Darwin 3, Madrid, 28049, Spain

**Keywords:** Embryo, Mouse, Storage

## Abstract

**Background:**

Interchange of genetically modified (GM) mice between laboratories using embryos provides several advantages. Not only is transport stress avoided, but also the health status of the recipient colony is not compromised. Embryos do not need to be shipped in frozen stage, which requires expensive packaging in addition to a certain degree of expertise in order to freeze and thaw them correctly. The aim of this study was to examine different storage conditions and their effect on embryo viability in order to establish the feasibility of practical, non-frozen conditions for embryo shipment.

**Methods:**

Mouse morulae developed *in vivo* (collected from donors 2.5d post coitum) or *in vitro* (zygotes cultured until morulae stage) were stored, combining two different media (KSOMeq or KSOM-H) and temperatures (4 degrees C, 15 degrees C and 37 degrees C) throughout 24 or 48 hours. After storage in vitro viability was assessed determining percentage of development to blastocyst and total cell number. In vivo viability was determined based on the number of implantations and living fetuses after embryo transfer of stored embryos. The storage effect at the molecular level was assessed by studying a gene pool involved in early development by quantitative RT-PCR.

**Results:**

*In vivo*-produced morulae stored for 24 hours did not show differences in development up to the blastocyst stage, regardless of the storage type. Even though a decrease in the total cell number *in vivo* was observed, embryo development after embryo transfer was not affected. All 24 hour storage conditions tested provided a similar number of implantations and fetuses at day 14 of pregnancy. Morulae obtained from *in vitro* embryo culture collected at the 1-cell stage showed a decreased ability to develop to blastocyst after 24 hours of storage at 15degrees C both in KSOMeq and KSOM-H. Concomitantly, a significant decrease of embryo implantation rates after transfer to recipients was also found. In order to further characterize the effect of non-frozen storage combining a molecular approach with the ordinary *in vitro* culture evaluation, embryos collected at the morula stage were submitted to the same storage conditions described throughout 48 hours. *In vitro* culture of those embryos showed a significant decrease in their developmental rate to blastocyst in both KSOMeq and KSOM-H at 15degrees C, which also affected the total number of cells. Gene transcription studies confirmed significant alterations in retrotransposons (*Erv4* and *Iap*) after 48 h of storage at 15degrees C.

**Conclusions:**

Our results show that both KSOMeq and KSOM-H can be equally used, and that several temperature conditions allow good survival rates *in vitro* and *in vivo*. Some of these storage conditions can substitute freezing in order to maintain embryo viability for 24–48 hours, providing a reliable and less demanding technical alternative for embryo interchanges.

## Background

An increasing number of genetically modified (GM) mice are exchanged between laboratories every year. National and international legislations enforce reducing the number of laboratory animals used for research purposes, and one clear way to accomplish this goal is to avoid the duplication of a GM line that has been already generated. Unfortunately, many of these lines are not stored in international repositories where cryobanking is well-established, but they are kept alive in animal facilities. Transport of live animals poses certain risks: not only may it compromise the health status of the receiving facility, but there is also an animal welfare issue and increasingly restrictive conditions from air and ground carriers.

On the other hand, cryopreserved embryos allow safe transport, but this approach demands a certain degree of expertise to freeze and thaw them correctly [1]. Moreover, the containers used are costly and should be returned with the concomitant expense. Some approaches have explored the feasibility of alternative methods using, for instance, intermediate recipients [2-4], or specific temperatures [5,6]. In these studies, 2-cell embryos were used, even though embryos of certain mice strains are prone to 2-cell blockage when cultured in suboptimal conditions [7]. A simple way to overcome this risk is to use embryos in later stages of development, like morulae, that still allow 48 hours of autonomy. This time span is enough to reach most destinations and can easily complement cryopreservation without a need for specific equipment and expertise in cryobiology. Transport of embryos in liquid stage can be achieved either in culture media or in standard embryo holding media like M-2, where bicarbonate is substituted with HEPES. With this replacement pH is stabilized when embryos are handled; however, bicarbonate is necessary for the optimal development of embryos and long-term exposure to holding media might be detrimental.

Our aim was to first explore different storage conditions, trying to simplify the technical skills needed in order to promote the use of embryos for exchanging GM lines, even with facilities unfamiliar with thawing procedures. Secondly, we tried to characterize how gene expression patterns of some relevant genes during preimplantational development are affected by storage conditions.

## Methods

### Reagents and Media

All chemicals and media were purchased from Sigma Chemical Co. (Madrid, Spain), unless otherwise stated.

### Embryo production

Female B6CBAF1 mice aged 6–8 weeks old were induced to superovulate with an intraperitoneal injection of 7.5 IU equine chorionic gonadotropin (eCG, Folligon, Intervet, The Netherlands), followed 48 h later, by 5 IU of human chorionic gonadotropin (hCG). Immediately after hCG administration, females were paired with adult intact mice of the same genetic background in a individual cage overnight. All animal experiments were approved by our Institutional Review Board according to the European and Spanish legislation.

### Embryo collection

One-cell embryos were collected from the ampulla of females which were euthanized by cervical dislocation, 20 h post-hCG administration. Cumulus cells were removed by exposure to hyaluronidase as described [8] and zygotes were washed in M2 medium and cultured in Potassium (K^+^) Simplex Optimized Medium (KSOM) microdrops [9] covered with mineral oil at 37°C and 5% CO_2_ until the morula stage. Morulae embryos developed *in vivo* were collected 66–78 h post-hCG administration by flushing both the oviduct and the upper part of the uterine horn with M2 medium.

### Storage conditions

Three temperatures (4°C, 15°C and 37°C) and two media (KSOM and KSOM-H) were chosen. Additionally, two storage lengths (24 and 48 hours) were studied in order to assess the effect of storage on in vitro development. Buffered media (KSOM-H) for embryo storage was prepared by adding HEPES (20 mM) to KSOM, maintaining bicarbonate at the original concentration of 25 mM. KSOM composition is shown at 23. Both media were prepared every 2 weeks and filtered through 0.22 μm pore and stored at 4°C until use.

**Figure 1:**
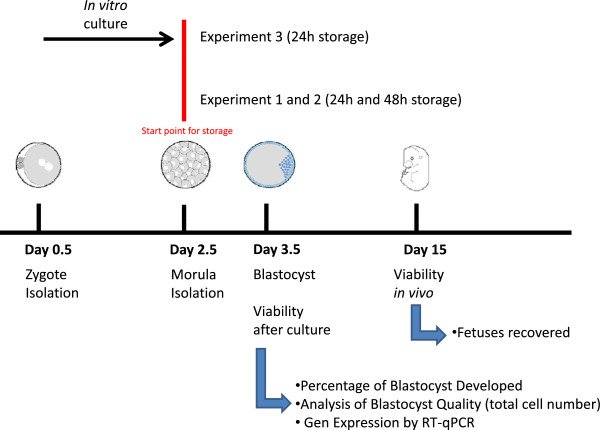
**Scheme of experimental design of the study.** Embryos cultured from zygote to morula stage were subjected to 24 hours of simulated shipment (Experiment 3). Embryos developed *in vivo* were subjected to either 24 hours (Experiment 1) or 48 hours (Experiment 2) of simulated shipment. In all cases, embryo development to blastocyst stage and total cell number was reordered. For 24 hour experiments, embryo transfer to surrogate mother foster was performed. Additionally, in the 48 hour experiment, gene expression analysis was carried out.

For experiments 1 and 2 of the *in vitro* study (Figure 1), twenty three B6CBAF1 females and eight B6CBAF1 males were used: morulae collected at day 2.5 dpc. were placed in cryotubes filled with pre-equilibrated KSOM (5 ml) for 24 h at 5% CO_2_ (KSOMeq) or KSOM-HEPES (5 ml, 20 mM HEPES, KSOM-H) at pH 7.3-7.4 and sealed with parafilm. Each tube contained 20–30 embryos; this high volume of medium allows a slow decrease in CO_2_ concentration and gradual changes in temperature. Randomly selected tubes were placed by couples (KSOMeq and KSOM-H), in a refrigerator (4°C), a refrigerated chamber (15°C) and in an incubator without CO_2_ (37°C). Twenty four hours (experiment 1) or 48 hours later (experiment 2), embryos were recovered from each tube and set in culture (KSOM microdrops). In each experiment, non-stored embryos were placed in culture in parallel to serve as a control group.

In experiment 3 (*in vitro* study), thirteen B6CBAF1 females and six B6CBAF1 males were used: 1-cell embryos collected on day 0.5 were placed in culture for 48–50 hours until reaching the morula stage. As described for the two previous experiments, groups of 20–30 embryos were placed in cryotubes filled with pre-equilibrated (24 h before) KSOM (5 ml) or KSOM-H and randomly assigned to one of the three storage conditions selected: 4°C, 15°C or 37°C. 24 hours later, embryos were recovered from each tube and placed in culture. For this experiment, a group of non-stored embryos obtained in the same way was set in culture as a control group for development.

### pH determination of holding media under the different storage conditions tested

In order to evaluate the pH fluctuations that might occur in equilibrated and buffered media used for embryo storage, pH measurements were performed on samples in triplicate, maintained under experimental storage conditions at 0, 90, 180 and 300 minutes, 24 h, 48 h and 72 h. for each treatment. Mean values were used as pH measurement at each time point.

### *In vitro* evaluation of viability of stored embryos

*In vitro* viability was evaluated in three different groups: *in vivo* collected morulae stored for 24 hours (experiment 1) or 48 hours (experiment 2) and morulae obtained from 1-cell embryos after *in vitro* culture and then stored for 24 hours (experiment 3). To perform these experiments, seventy-five B6CBAF1 females were used as embryo donors, seventy-three CD-1 females as surrogate mothers; ten B6CBAF1 males and ten CD1 males were used to establish matings. In all cases, embryos were randomly distributed into the six combinations of media and temperature described, plus into a control group of cultured embryos without previous storage. Two parameters were determined: percentage of development to blastocyst, and total cell count. To determine the *in vitro* viability based on development to blastocyst, embryos were recovered after storage from cryotubes and placed in pre-equilibrated KSOM microdrops. After incubation at 37°C and 5% CO2 for 24–36 h, the number of expanded blastocysts was recorded.

The total cell number of blastocyst which had developed under the different storage conditions and in control groups was determined by nuclei staining. Embryos were placed in M2 medium drops supplemented with Hoechst 33342 (1 *μ*g/ml) and allowed to stain during 30 minutes at 37°C. Blastocysts were placed on a glass slide, sealed with a cover slide and observed under a Nikon Optiphot-2 fluorescence microscope with an appropriate filter set (Blue: 450-490 nm excitation filter, 515 nm barrier filter).

### *In vivo* evaluation of viability of stored embryos

Embryos stored under the different conditions were, immediately after the storage period, surgically transferred to surrogate mothers in order to establish their *in vivo* viability. 15 to 20 embryos recovered from the different treatments were transferred into the left oviduct of 0.5 pseudopregnant CD-1 females (Harlan), plugged by vasectomised males. At day 14 of gestation, recipients were euthanized and uterine horns were isolated to record the number of living fetuses, resorptions and the total number of implantations.

### Quantitative RT-PCR

Gene expression studies were performed on *in vivo* collected morulae stored for 48 hours in order to maximize effects of storage without interference of previous *in vitro* culture. The quantification of all gene transcripts was carried out by real time quantitative RT-PCR in three replicates (10 embryos per replicate) [10]. A total of 210 blastocysts were used; sixteen B6CBAF1 females and six B6CBAF1 males were used to collect the embryos. PCR was performed using a Rotorgene 2000 Real Time Cycler™ (Corbett Research, Sydney, Australia) and SYBR Green (Molecular Probes, Eugene, OR) as a double-stranded DNA-specific fluorescent dye. The PCR reaction mixture (25 μl) contained 2.5 μl 10× buffer, 3 mM MgCl_2_, 2 U Taq Express (MWGAG Biotech, Ebersberg, Germany), 100 μM of each dNTP, and 0.2 μM of each primer (Table 1). In addition, the double-stranded DNA dye, SYBR Green I, (1:3000 of 10000× stock solution) was included in each reaction. The PCR protocol included an initial step of 94°C (2 min), followed by 40 cycles of 94°C (15 s), 56–59°C (30 s) and 72°C (30 s). Fluorescent data was acquired at 80–85°C. The melting protocol consisted of holding at 40°C for 60 s, and then heating from 50 to 94 C, holding at each temperature for 5 s while monitoring fluorescence. Product identity was confirmed by ethidium-bromide-stained 2% agarose gel electrophoresis. As negative controls, tubes were prepared in which RNA or reverse transcriptase was omitted during the RT-reaction. The comparative CT method was used for quantitation of expression levels. The quantification was normalized to the endogenous control histone H2a.z. The normality of amplification was checked for each sample. The internal control is used to normalize the PCRs for the amount of RNA added to the reverse transcription reactions and to minimize variability in the results due to differences in the RT efficiency and/or RNA integrity among the different embryo groups. The CT value obtained for each amplified gene was normalized to the CT value of an internal control, histone H2a.z. Within this region of the amplification curve, each difference of one cycle is equivalent to a doubling of the amplified product of the PCR. According to the comparative CT method, the ΔCT value was determined by subtracting the H2a CT value for each sample from each gene CT value of the sample. Calculation of ΔΔ CT involved using the highest sample ΔCT values (i.e. the sample with the lower target expression) as an arbitrary constant to subtract from all other ΔCT sample values. Fold changes in the relative gene expression of the target was determined by using the formula, 2-ΔΔCT [11].

**Table 1:** **Total cell counts of blastocysts developed after morulae (*in vivo *developed) storage during 24 hours**

**Treatment**		**Number of blastocysts analysed**	**Total cell (N° of cells)**
**Media**	**Temperature**		
Control	37°C	19	59.26 ± 2.86 ^a^
KSOM	4°C	26	40.77 ± 1.96 ^b^
KSOM-HEPES		17	48.94 ± 2.67 ^b^
KSOM	15°C	21	40.24 ± 2.05 ^b^
KSOM-HEPES		19	44.58 ± 2.12 ^b^
KSOM	37°C	21	46.10 ± 3.11 ^b^
KSOM-HEPES		22	50.46 ± 3.93 ^b^

Mean ± SEM. Different letters indicate significant differences between treatments. ANOVA followed by Student-Newman-Kleus post hoc test was performed.

### Experimental design

Our aim was to assess viability of embryos stored in different conditions that could be easily used for transport without requirement of specific containers or specialized recovery techniques. Temperatures were selected to include those whereat embryos could be in a latent stage (4°C) [12], or at a physiological developmental temperature (37°C) [13,14]. An additional temperature was selected whereat metabolism could be retarded and easily maintained without complex equipments (15°). These temperatures were combined with 2 different media: KSOMeq and KSOM-H. Three different studies were designed to achieve this goal. In the first study (Figure 1) we assessed *in vitro* viability after storage in three experiments: In experiment 1, freshly collected morulae were stored in different conditions for 24 hours. In experiment 2, the same storage conditions were tested for 48 hours, and in experiment 3, embryos were collected at the 1-cell stage, cultured *in vitro* until the morula stage and then submitted to the same storage conditions for 24 hours as in experiment 1. *In vitro* viability was determined based on percentage of development in culture and assessing total cell number.

In the second study, in *vivo* viability of stored embryos was evaluated based on the number of live implantations and resorptions after being transferred into foster mothers. *In vivo* assessment of viability was circumscribed to embryos stored for 24 h; either freshly collected morulae or morulae obtained after *in vitro* culture of embryos collected at the 1-cell stage, since in those groups, conditions were represented that, *in vitro,* were either detrimental or did not affect embryo development.

The third study explored the effect of storage conditions on expression of some genes closely related to early embryo development. To determine these levels of gene expression, we chose embryos obtained *in vivo* stored for 48 h under the different conditions studied. We selected this time condition in order to examine gene expression after storage in those combinations of temperature and media in which a relevant effect was assessed. Correlation between gene expression and physiological aspects (embryo development in culture) allows for defining new molecular aspects of embryo physiology.

### Statistical analysis

Statistical analyses were performed using SigmaStat version 3.1.1 software (Jandel Scientific, San Rafael, CA). Data is given as the mean ± S.E.M. Comparison of the differences between means for each treatment were done using ANOVA followed by Student-Newman-Kleus post hoc test.

## Results

### *In vitro* development after storage for 24 or 48 hours

**Figure 2:**
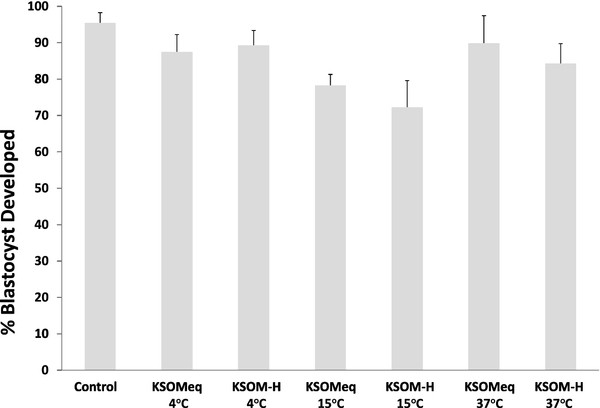
**Development of embryos collected at the morula stage stored for 24 hours in KSOM and KSOM-HEPES at 4°C, 15°C and 37°C.** Mean ± SEM. Significant differences were denoted by different letters. ANOVA analysis was performed.

None of the 24 hours storage conditions evaluated (Figure 1.) affected *in vitro* development of embryos collected at the morula stage. Almost 100% of morulae assigned to the control group (submitted to culture without previous storage) developed to blastyocyst (Figure 2.) Among those morulae stored for 24 hours in the 6 conditions tested, one particular storage condition (KSOM-H at 15°C) decreased percentage of blastocyst formation to 72%, but this decrease was not statistically significant.

**Figure 3:**
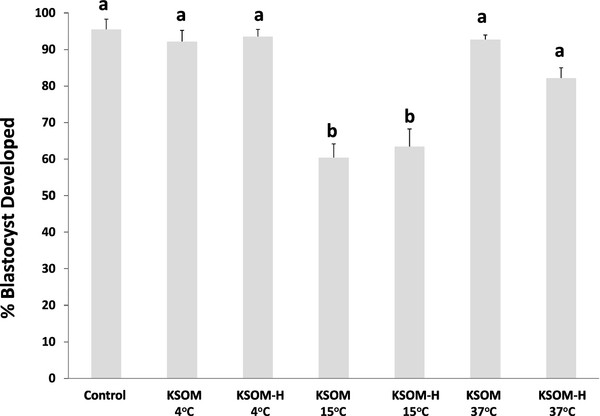
**Development of embryos collected at the morula stage (*in vivo *developed) and stored for 48 hours in KSOM and KSOM-HEPES at 4°C, 15°C and 37°C.** Mean ± SEM. Significant differences were denoted by different letters. ANOVA analysis was performed.

When storage time is extended to 48 hours, embryos stored at 4°C (both KSOMeq and KSOM-H treatments) showed the highest level of development, but these values were statistically similar to those found in embryos stored at 37°C, regardless of the medium used. Embryo development decreased to 60% in both 15°C treatments (Figure 3).

**Figure 4:**
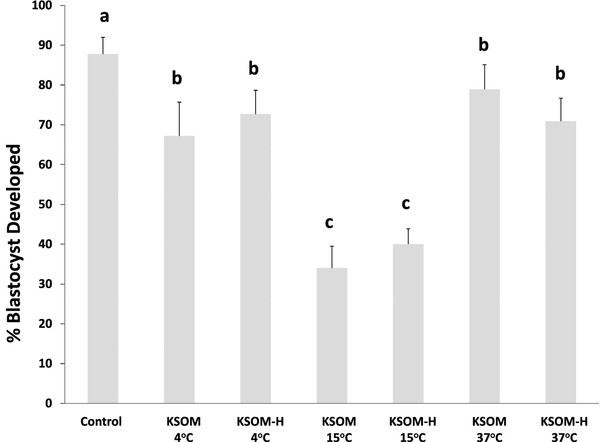
**Development of embryos collected at 1-cell stage, cultured to morulae (*in vitro *developed) and stored for 24 hours in KSOM and KSOM-HEPES at 4°C, 15°C and 37°C.** Mean ± SEM. Significant differences were denoted by different letters. ANOVA analysis was performed.

In order to examine the effect of *in vitro* culture on morulae embryos’ ability to be stored under several conditions, a pool of zygotes was retrieved on Day 1 and placed in culture for 60 hours until reaching the morula stage (Figure 1). Then, embryos were allocated to the six storage conditions described for the rest of experiments, and a group remained in culture to serve as control. Embryo viability was significantly compromised under two of the conditions tested (KSOMeq and KSOM-H at 15°C). While the control group showed developing rates close to 90%, a significant decrease in viability was observed for embryos stored at 15°C, regardless of the medium selected. Blastocyst development dropped to 30% in KSOMeq and to 40% in KSOM-H. In the remaining groups, no differences were evidenced when compared to the control. Embryos stored at 4°C (both KSOMeq and KSOM-H) showed developing rates close to 70%, while embryos stored at 37°C showed viability rates close to 80% and 70% (KSOMeq and KSOM-H, respectively) (Figure 4 ).

### Total cell number

Correlation between the cell number in the blastocyst, and implantation ability was reported several years ago [15]. However, this parameter correlates poorly with morphology, a visual character widely used as a non-invasive marker for embryo quality. For this reason, morphological criterion should be reinforced by other parameters [16]. In addition to morphology, we analyzed the total cell number of morulae developed *in vivo* subjected to storage during 24 hours. As a result of this study, we observed a significant cell number decrease in all treatments in comparison with the control group (Table 1), but no differences were found when we compared this parameter within the different experimental conditions tested.

*In vivo* developed morulae that were stored for 48 hours showed a significant decrease in cell count compared to the control group in both media (KSOMeq and KSOM-H) in embryos stored at 15° and 37°C (Table 2.). Conversely, 4°C treatments did not differ. When cell numbers were compared within the two groups at 37°C, KSOMeq provided higher values than KSOM-H, suggesting a detrimental effect of HEPES at such a temperature (Table 3).

**Table 2:** **Total cell count of blastocysts developed after morulae (*in vivo *developed) storage during 48 hours**

**Treatment**		**Number of blastocysts analysed**	**Total cell (N° of cells)**
**Media**	**Temperature**		
Control	37°C	23	69.43 ± 3.19 ^a^
KSOM	4°C	15	64.67 ± 1.97 ^a^
KSOM-HEPES		21	70.05 ± 2.80 ^a^
KSOM	15°C	14	41.14 ± 2.66 ^b^
KSOM-HEPES		23	43.30 ± 2.12 ^b^
KSOM	37°C	39	52.97 ± 1.39 ^c^
KSOM-HEPES		22	38.15 ± 1.47 ^b^

Mean ± SEM. Different letters indicate significant differences between treatments. ANOVA followed by Student-Newman-Kleus post hoc test was performed.

**Table 3:** **Total cell counts of blastocysts developed after morulae (*in vitro *developed) storage during 24 hours**

**Treatment**		**Number of blastocysts analysed**	**Total cell (N° of cells)**
**Media**	**Temperature**		
Control	37°C	19	54.73 ± 1.74^a^
KSOM	4°C	24	40.00 ± 1.98^b^
KSOM-HEPES		16	44.88 ± 2.38 ^b^
KSOM	15°C	18	43.63 ± 1.85 ^b^
KSOM-HEPES		13	32.61 ± 3.08 ^c^
KSOM	37°C	18	52.39 ± 2.97^a^
KSOM-HEPES		15	32.83 ± 2.96 ^c^

Mean ± SEM. Different letters indicate significant differences between treatments. ANOVA followed by Student-Newman-Kleus post hoc test was performed.

*In vitro* developed morulae showed a significant decrease in cell number in all treatments, except in KSOM at 37°C, with the highest decrease found in KSOM-H at 15°C and at 37°C. This fact indicates that media alkalinization proved to be less harmful than HEPES presence, since we found a significant difference in this temperature (52.39 vs 32.83 (KSOM at 37°C vs KSOM-H at 37°C)). This effect was not present in those embryos collected as morulae and subjected to the same storage conditions as those described in experiment 1 (Table 3). This suggests that the coincidence of HEPES presence was necessary with other detrimental conditions in order to produce a measurable negative effect.

### *In vivo* viability of stored embryos: analysis of implantations and fetuses

This study was carried out with embryos stored for 24 h, either freshly collected morulae or morulae obtained after an *in vitro* culture of embryos collected at the 1-cell stage. *In vivo* viability was measured by determining the number of live implantations and resorptions after embryo transfer into foster mothers. Table 4 summarizes the *in vivo* viability of embryos collected at the morula stage and stored for 24 hours in the six conditions assayed, compared to a control group of transfers performed with embryos collected at the morula stage and transferred without further storage. It may be observed that none of the storage conditions produced a significant decrease in implantation percentages compared to the control. Values oscillated between 41% (KSOMeq at 15°C) to 66% (KSOM-H at 15°C), without significant differences. However a significant raise of resorptions was observed in the KSOMeq at 15°C (38%) compared to the 16% registered in the control group.

**Table 4:** **Number of fetuses, resoprtions and total number of implantations after embryo transfer of morulae (*in vivo *developed) stored during 24 hours**

**Treatments**		**Number of experiments**	**Embryos transferred**	**Fetuses (%)**	**Resorptions (%)**	**Total implantations (%)**
**Media**	**Temperature**					
Control	37°C	5	70	41 (58.48 ± 7.76) ^a^	11 (15.90 ± 4.98)^nd^	52 (74.37 ± 6.83)^nd^
KSOM	4°C	5	77	38 (49.59 ± 9.57) ^a^	16 (21.15 ± 8.23)	54 (70.75 ± 13.63)
KSOM-HEPES		5	73	30 (42.92 ± 5.55) ^a^	15 (32.34 ± 7.41)	45 (75.26 ± 6.18)
KSOM	15°C	5	80	33 (41.05 ± 4.02) ^a^	31 (38.70 ± 4.63)	64 (79.75 ± 5.31)
KSOM-HEPES		5	71	47 (66.29 ± 2.79) ^a^	14 (20.19 ± 3.57)	61 (86.47 ± 4.72)
KSOM	37°C	5	78	41 (52.95 ± 9.97) ^a^	18 (22.16 ± 6.41)	59 (75.11 ± 10.62)
KSOM-HEPES		5	78	44 (56.21 ± 6.28) ^a^	19 (24.01 ± 10.22)	63 (80.23 ± 5.81)

Mean ± SEM. Different letters indicate significant differences between treatments. ANOVA followed by Student-Newman-Kleus post hoc test was performed.

Survival of control embryos collected at the 1-cell stage, cultured to the morula stage and then transferred into pseudopregnant recipients without storage, was 24% as opposed to the 41% of live fetuses obtained after transfer of embryos collected as morulae and immediately transferred. This difference highlights the detrimental effect of *in vitro* culture on viability. All treatments provided a similar number of fetuses recovered on day 14 compared to the control group, with the exception of KSOM-H at 15°C. In this case, the percentage of live fetuses decreased to 5%. This same group showed a significantly higher resorption. However, there were no differences between treatments regarding the total number of implantations (Table 5). These values suggest that the embryo’s ability to implant is preserved, but that ICM cells are not able to develop into fetuses, indicating a possible misregulation of genes related to post-implantation development.

**Table 5:** **Number of fetuses, resoprtions and total number of implantations after embryo transfer of morulae (*in vitro *developed) stored during 24 hours**

**Treatments**		**Number of experiments**	**Embryos transferred**	**Fetuses (%)**	**Resorptions (%)**	**Total implantations (%)**
**Media**	**Temperature**					
Control	37°C	5	82	24 (30.6 ± 2.7)^a^	19 (23.6 ± 4.09)^a^	43 (57.5 ± 5.74)^n.d.^
KSOM	4°C	5	92	31 (32.8 ± 6.38)^a^	25 (29.8 ± 8.96)^ab^	56 (62.59 ± 5.78)
KSOM-HEPES		5	77	21 (29.5 ± 6.38)^a^	26 (34.5 ± 5.27)^ab^	47 (64.07 ± 6.90)
KSOM	15°C	6	86	11 (13.6 ± 3.99)^ab^	35 (40.4 ± 6.80)^ab^	46 (53.96 ± 8.62)
KSOM-HEPES		5	80	4 (4.67 ± 2.34)^b^	47 (63.9 ± 13.4)^b^	51 (69.94 ± 12.89)
KSOM	37°C	6	90	12 (16.4 ± 5.60)^ab^	40 (45.2 ± 6.35)^ab^	52 (60.49 ± 9.15)
KSOM-HEPES		6	98	17 (17.0 ± 3.76)^ab^	36 (37.8 ± 8.40)^ab^	53 (54.81 ± 9.48)

Mean ± SEM. Different letters indicate significant differences between treatments. ANOVA followed by Student-Newman-Kleus post hoc test was performed.

### Transcription analysis by real time PCR

Since our findings suggested a certain misregulation of the genes crucial for embryo development after implantation, we evaluated the effect of storage on mRNA expression. For that purpose, we chose *in vivo* collected morulae stored for 48 h. Gene screening aimed to check different developmental functions (Table 6). *Bax* versus *Bcl-2* ratio was used as prognostic marker of apoptosis. None of the assayed conditions altered the expression level of *Bax*, a proapoptotic gene. On the contrary, *Bcl-2*, an antiapoptotic gene, was downregulated in all experimental conditions compared to the control and, as a consequence, *Bax*/*Bcl-2* ratio was altered in all treatments assayed when compared to the control group. This indicates the occurrence of apoptotic events during storage (Figure 6A).

**Table 6:** Details of primers used for RT-PCR

**Gen symbol**	**MGI official name**	**Primers sequence (5**^**′**^**– 3**^**′**^**)**	**Fragment size**	**Gene bank accesion n°**
Bax	BCL2-associated X protein	5^′^ CTACTTTGCCAGCAAACTGG	159	NM_007527.3
		3^′^ TCCCAAAGTAGGAGAGGA		
Bcl2	BCL2-like 1	5^′^ GGAGCTGGTGGTTGACTTTC	517	NM_009743.4|
		3^′^ CTAGGTGGTCATTCAGGTAAG		
ErV4	Murine endogenous retovirus-L	5^′^ TGCTTGGGCTCAGCAACATGG	278	XM_001478088.1|
		3^′^ GACAGAATGCCTCATCTATCGT		
Iap	Intracisternal-A particle	5^′^ GGGTATTGTTGAGCGTGCGC	333	XM_001477167.1|
		3^′^ TCGGGTGAGTCTTTCTGGTAC		
Terf1	Telomeric repeat binding factor 1	5^′^ TTCAACAACCGAACAAGTGTC	215	Mm 4306
		3^′^TCTCTTTCTCTTCCCCCTCC		
Cx43 (Gaj1)	Gap junction membrane channel protein alpha 1	5^′^TACCACGCCACCACTGGCCCA	290	Mm 4504
		3^′^ATTCTGGTTGTCGTCGGGGAAATC		
Nanog	Nanog homebox	5^′^AGGGTCTGCTACTGAGATGCTCTG	363	Mm 6047
		3^′^CAACCACTGGTTTTTCTGCCACCG		
Oct3/4 (Pou5f1)	POU domain, class 5, transcription factor 1	5^′^GGAGAGGTGAAACCGTCCCTAGG	312	Mm 17031
		3^′^AGAGGAGGTTCCCTCTGAGTTGC		
Gapdh	Glyceraldehyde-3-Phosphate dehydrogenase	5^′^GGGTGTGAACCACGAGAAATATGA	250	Mm 379644
		3^′^CCTTCCACAATGCCAAAGT		
H2afz	Histone H2az	5^′^TGTGTACAGCGCAGCCATCCTG	208	NM_016750.2
		3^′^CTTCCCGATCAGCGATTTGTGG		

Retrotransposon expression, frequently impaired in stressful embryo situations, was analysed measuring mRNA levels of *Erv4* and *Iap* genes. *Erv4* was significantly upregulated in KSOMeq and KSOM-H at 15°C, Furthermore, *Iap* showed a marked increase in mRNA levels in embryos kept in KSOM-H at 5°C when compared to controls. These changes in retrotransposon transcription indicate some alterations in the epigenetic control of their expression. While no effect was detected in the expression of reverse transcriptase for telomerase (*Tert*), *Gaj1* (Connexin 43) showed a decrease in levels of transcripts at 15° and 37°, both in KSOMeq and in KSOM-H. This situation could have detrimental effects on the implantation of stored embryos, advising against the use of these temperature conditions for embryo transport (Figure 6D).

Nanog transcript levels decreased in all treatments compared with the control group, but the effect was especially remarkable in KSOM and KSOM-H at 15° and 37°C. A similar pattern had *Pou5F1* (Oct 3/4), decreasing transcripts in KSOM at 15°C and both KSOM as KSOM-H at 37°C (Figure 6C).

*Gapdh* is a gene involved in metabolic functions, such as in transcription and programmed cell death. In this case, it had a slightly significative tendency to be downregulated at higher temperatures. KSOM at 15C and KSOM-H at 37°C were the groups in which downregulation was more evident (Figure 6B).

### Holding medium pH fluctuation during storage

**Figure 5:**
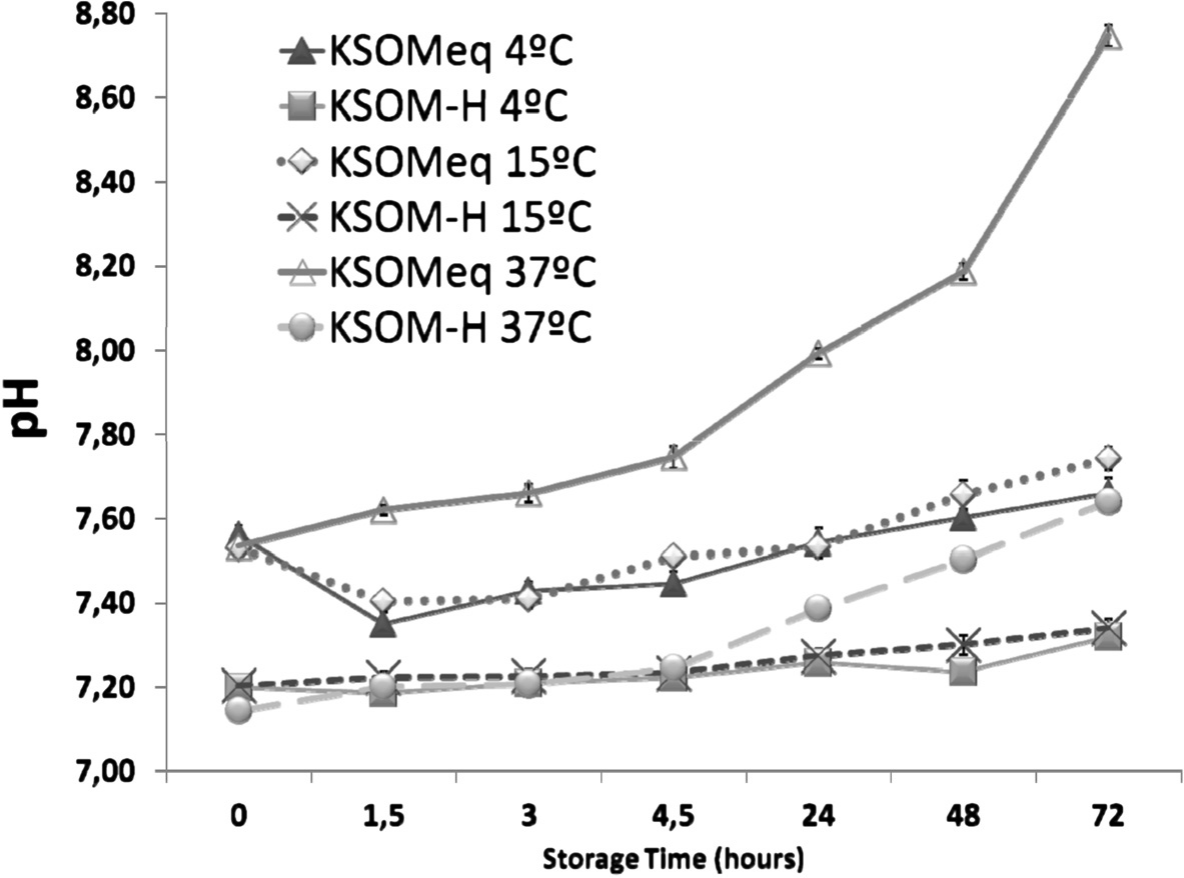
Media pH changes during storage of embryos under different conditions assayed.

Figure 5 summarizes pH media fluctuation. All experimental conditions provided initial pH under 7.6. However after keeping equilibrated KSOM at 37°C for 48 hours, media underwent a consistent basification reaching values close to 8, demonstrating that the buffering activity of bicarbonate could not guarantee pH conditions, even in a tight sealed vial.

**Figure 6:**
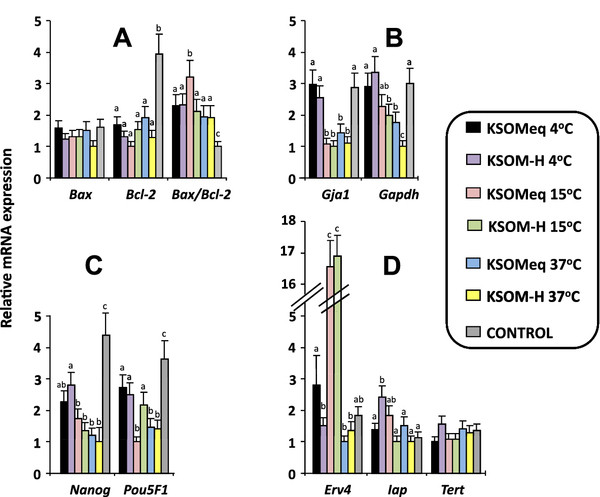
**Analysis of mRNA transcription levels for developmentally important genes.** Genes assayed were: A) apoptosis (*Bax*, *Bcl2*), B) cell-to-cell adhesion (*Gaj1*), and methabolism (*Gapdh*) C) totipotency (*Pou5f1*, *Nanog*) D) retrotransposon expression (*Erv4* and *Iap*) and telomere extension (*Terf1*), after storage for 48 hours in KSOM and KSOMh at 4°C, 15°C and 37°C. Mean ± SEM. Significant differences were denoted by different letters. ANOVA analysis was performed.

Since our intention was to test standard recipients within the reach of any laboratory, we performed the study in cryotubes. These recipients close tightly and provide a theoretical way to isolate the growth media in which embryos could be stored. Analysis of pH revealed a significant difference in pH values of KSOMeq at 37°C in comparison with the the rest of treatments. These findings demonstrated that cryotubes allow CO_2_ difussion into the air and a concomitant basification that was not buffered by the presence of HEPES as in KSOM-H, and that this diffusion was potentiated by temperature [17]. More efficient ways to preserve storage environment have been reported [18], that should be considered as an alternative if transport has to take place under such conditions.

## Discussion

Shipment of embryos is a good alternative to the exchange of live animals for several reasons. The intrinsic sterility of embryos surrounded by an intact zona pellucida obviates differences in the microbiologic levels of donor and recipient laboratories [19], [20]. Transport of embryos also has the advantage of avoiding stressful conditions that may affect animals for several days. In addition, use of embryos when dealing with genetically modified animals avoids any possibility of accidental escape, an important issue in risk assessments. Embryos of different mammalian species have been successfully transported in frozen stage [21-23]. Cryopreserved embryos are a reliable method and they can reach any remote location in the world; conversely, fresh embryos remain viable for a limited amount of time and they require careful coordination between sending and receiving facilities. However, they allow the use of simple containers instead of dry shippers and, what is more important: they do not require cryopreservation skills to correctly freeze and thaw embryos.

These advantages have motivated several studies to set up non-frozen transport conditions [24-26]. In most cases, they were circumscribed to the 2-cell stage. The reason to choose this specific developmental stage may rely on providing the more extended length of *in vitro* culture for positively fertilized embryos. However, this stage poses a risk in certain strains, since 2-cell block mostly appears linked to suboptimal culture conditions [7,27-29].

We wanted to define more universal conditions by exploring different temperatures and the role of pH, comparing an equilibrated versus a HEPES buffered medium. We also chose morula stage, a less restrictive stage since the 2-cell block has already been overcome and there are still 24–48 hours of autonomy. In addition, we explored the feasibility of collecting embryos at 1-cell stage and culturing them to morulae before storage, since collecting embryos from the ampulla is consistently easier than flushing them from the oviduct, and our aim was to define the most accessible way to ship embryos under non-frozen conditions.

Our results in the first experiment demonstrate that *in vitro* development after 24 hours of storage under the conditions described is not compromised. Our findings showed that, even though there was a significant decrease of cell counts in all treatments with respect to the control group, this did not compromise *in vivo* viability after transfer. Somehow, the certain delay in development due to storage, which motivates the decrease in total cell numbers in stored embryos, was easily overcome when they were placed in a permissive maternal environment, such as a 0.5 recipient. This demonstrates that successful transfer of morulae after 24 h of storage can be achieved in any of the media or temperature assayed without compromising *in vivo* viability. Previous studies have focused on the transport of 2-cell embryos, either thawed after cryopreservation, [30] or obtained from *in vitro* fertilization [5], achieving 44% *in vivo* development for embryos stored at 4°C for 48 hours. We obtained higher developmental levels under all the conditions tested, but in our study, embryo transfer was performed after 24 hours of storage. Given the significant decrease of *in vitro* viability observed when comparing 24 to 48 hours of storage, and the detrimental effect of *in vitro* embryo culture previous to being subjected to storage conditions, we can assume that for longer storage periods, 4°C is more advisable. At 15°C or 37°C, the induced gene deregulation and the decrease in cell count may not be compensated by the maternal environment after transfer, as occurred with 24 h stored embryos.

The *in vivo* and *in vitro* alterations observed after the storage 48 h may be a consequence of the misregulation of genes with an important role in embryo development. For example, the low percentage of blastocyst development after storage at 15°C can be related to the increase in deregulation in *Bax/Bcl-2* ratio and the increase in the retrotrasposon expression observed in this group, since *Bcl2 Bax* ratio has been considered a marker of viability or apoptosis in bovine embryos [31].

The cell number decrease observed both in the 15°C and 37°C groups could be related to the decrease in *Nanog* and *Oct3/4* expression. The expression patterns we found might also suggest a decrease in the pluripotency ability of ICM cells and, to some extent, may be responsible for the failure in postimplantation development found after transfer of stored embryos at 15°C [32,33]. Expression patterns in the group of embryos stored at 4°C, were more similar to the patterns observed in the control group, which could also explain the higher percentage of living fetuses obtained after transfer of embryos collected at 1-cell and in vitro cultured prior to storage.

Although several studies have demonstrated embryo ability to develop after long periods under non-physiological conditions [6,34] , we did not intend to analyze culture conditions. Differences between those analyses and the findings presented here are relevant. We focused on defining a way to store embryos from a practical point of view; for this reason, we compared several temperatures instead of focusing only on one. On the other hand, since long- term transport is successfully achieved with frozen embryos, we focused on short-term conditions. Nowadays, most courier transports deliver in 24–48 hours. Therefore the aim of our study was not to examine in depth what the limit for survival of an embryo is under different conditions, but rather if any of the conditions analysed could be addressed as the optimal one for short-term shipment.

Standard embryo holding media, such as M-2 substitute bicarbonate with HEPES, are useful in order to stabilize pH when embryos are handled; however, bicarbonate is necessary for the optimal development of embryos [34] while being kept at 4°C. Buffering is not the only role of NaHCO_3_, since CO_2_ is required for in vitro embryo development. Carbon from external CO_2_ is fixed by the embryo and used for various metabolic processes [35,36]. Some authors consider that buffer mixtures as HEPES-MOPS together with 25 mM NaHCO_3_ are enough to maintain pH [18].

With this in mind, in our experimental design, bicarbonate was not removed from the medium. KSOM-H consisted of the same standard KSOM medium with 25 mM sodium bicarbonate, supplemented with HEPES, in order to provide pH buffering ability without reducing the amount of available bicarbonate.

The volume in which embryos were contained is an important parameter. Mouse embryos are sensitive to osmolarity increase [37]; due to this circumstance, and so as to avoid evaporation during storage time, a large volume for the medium was chosen. In addition, pH buffering is more accurate in large volumes, especially in KSOMeq. For this reason, storage conditions should be considered as a whole, since volume may influence gas interchange and pH fluctuation.

In this study, most treatments implied a significant reduction in cell numbers in comparison with the control, but these differences did not affect implantation ability [38]. Reduced cell numbers per embryo do not always indicate a decline in the subsequent in vitro or *in vivo* developmental capacity of embryos, as some authors have reported in other processes, such as cryopreservation [39]. Some theories have been proposed, but essentially, embryos with reduced numbers of cells are still able to give rise to fetuses [40-42]. Nowadays, is not fully understood what the limit is to this situation. It is proposed that ICM cells have an important role in the three germ layer formation of the embryo. If too small, ICM is present, so embryonic endoderm cannot be formed. Although only the embryonic ectoderm gives rise to the embryo proper, the interactions between the three germ layers are necessary for full embryonic and fetal development.

Our aim was to define the easiest way possible to obtain and store embryos for a short time, eliminating the need for cryopreservation. For this reason, in our study, we also tested the feasibility of using morulae obtained after *in vitro* culture of embryos collected at the 1-cell stage. One cell embryo collection is technically easy, and *in vitro* culture avoids the necessity of correct identification of fertilized embryos, which requires a certain skill. However, this approach proved to be less efficient. Even though live fetuses were obtained from all storage conditions, there was a sensitive increase in the number of resorptions. This demonstrated that embryos had a compromised viability; they could be implanted, but were incapable of further development. Our results regarding cell counts somehow illustrate that situation: morulae obtained *in vitro* had a lower total cell number in comparison with the numbers observed on *in vivo* collected morulae. Even though embryos with reduced cell numbers are able to implant and develop, a low cell count has been associated with lower developmental ability [43].

Our results show that morula is a very permissive developmental stage for 24 h embryo storage, allowing different temperatures and media conditions without detrimental effects on *in vivo* viability after transfer. Even though previous *in vitro* culture seriously compromises embryo viability, this effect is more dramatic when storing under suboptimal conditions, such as 15°C where deregulation of important genes became highly significant. But even under such conditions, it was possible to obtain live fetuses after transfer. Since none of the 24 h storage conditions tested showed a significant improvement or a deleterious effect, there is no scientific basis to choose one or other. Practical preferences are a valid criterium to decide on a given temperature. However, if it is suspected a certain delay in the process or previous culture of embryos is necessary, our results based on the higher implantation rates found, suggest that 4°C provides a safer margin.

## Conclusions

In conclusion, both KSOMeq and KSOM-H may be equally used for embryo storage, and several temperature conditions allow for good *in vitro* and *in vivo* survival. Some of these storage conditions can substitute freezing in order to maintain embryo viability for 24–48 hours, offering a good and technically less demanding alternative for embryo exchanges.

## Competing interests

The authors declare that they have no competing interests.

## Authors' contributions

Experiment conceived and designed by BP, JDH and AGA. Experiment performed by JDH, AS, MPC and BP. Data analyzed by JDH, BP and AGA. Paper discussed and written by JDH, BP, AGA and MPC. All authors read and approved the final manuscript.
